# SLEEP quality in patients with psoriatic arthritis and its relationship with disease activity and comorbidities: a cross-sectional study

**DOI:** 10.1038/s41598-023-48723-z

**Published:** 2023-12-21

**Authors:** Esther Toledano, Cristina Hidalgo, Luis Gómez-Lechón, Marta ibáñez, Carolina Cristina Chacón, Javier Martín-Vallejo, Sonia Pastor, Carlos Montilla

**Affiliations:** 1 Rheumatology Department, Hospital Universitario Clínico San Carlos, Madrid, Spain; 2 Rheumatology Department, Hospital Universitario de Salamanca, Salamanca, Spain; 3 Rheumatology Department, Hospital Francesc De Borja, Gandía, Valencia Spain; 4 Statistics Department, Universidad de Salamanca, Salamanca, Spain

**Keywords:** Psoriatic arthritis, Sleep disorders

## Abstract

The assessment of psoriatic arthritis is complex and multidimensional. It is increasingly common to include the patient perspective using patient-reported outcomes. Although some research has explored sleep quality in patients with psoriatic arthritis, most studies have had small sample sizes, failed to assess sleep quality considering the inflammatory process together with the psychological well-being of patients, and have not described any use of sleep medication. Further, research to date has not provided data on the relationship of sleep quality with axial forms. In this context, the objective of this study was to assess sleep quality in patients with psoriatic arthritis and its relationship with clinical characteristics, disease activity, functioning, disease impact, fatigue and psychological status. A cross-sectional study was conducted including 247 consecutive patients with PsA recruited during 2021. Sleep quality was measured using the Pittsburgh Sleep Quality Index. We assessed correlations of Pittsburgh Sleep Quality Index score with peripheral disease activity (Disease Activity Index for PSoriatic Arthritis), axial disease activity (Ankylosing Spondylitis Disease Activity Score-C-reactive protein and Bath Ankylosing Spondylitis Disease Activity Index), functioning (Bath Ankylosing Spondylitis Functional Index and Health Assessment Questionnaire), impact (Psoriatic Arthritis Impact of Disease questionnaire), anxiety, depression (Hospital Anxiety and Depression Scale) and fatigue (Functional Assessment of Chronic Illness Therapy-Fatigue) scores. A multiple linear regression model was constructed with PSQI as the dependent variable and as independent variables those that could influence sleep quality. Nearly two-thirds (63.15%) of patients had poor sleep quality. Poorer sleep quality was associated with being female, higher joint counts, greater peripheral and axial disease activity, fatigue, anxiety and depression, functioning and disease impact (p < 0.001). Multiple linear regression analysis found that pain (β: 0.3; p < 0.007) and fatigue β: − 0.1; p < 0.001 contributed 40% to the sleep quality model. Poor sleep quality was common among patients with psoriatic arthritis. Emotional factors (fatigue, anxiety) seemed more important than inflammatory factors in sleep quality.

## Introduction

Psoriatic arthritis, a member of the spondyloathritis family of diseases, is a chronic inflammatory disease associated with psoriasis. According to data from the study of prevalence of rheumatic diseases in the Spanish adult population (EPISER 2016)^1^, the prevalence of this disease is 0.52 (CI 0.38–0.87%).

Apart from axial and peripheral joint involvement, the clinical description of PsA includes entheseal changes and dactylitis as well as extra-articular manifestations. In any of its manifestations, psoriatic disease is associated with impairment of social functioning and psychological disorders^2^.

The practice of assessing the psychological well-being of patients with PsA is becoming increasingly common and systematic. A recent study obtained, in a Spanish population, a prevalence of sleep disorders of 21.1% (CI 95% 17.38–25.01)^3^. The impact of the disease on sleep quality has been well documented previously^4–8^. This psoriatic disease can have a significant effect on the feeling of fatigue^9^ as well as impair sleep quality with respect to that in the general population^10^. In turn, a reduction in sleep quality is related to a decrease in the quality of life^11,12^ and the development of comorbidities^13–16^. The persistence and severity of sleep disorders may be associated with inflammatory disease activity, chronic pain, fatigue, anxiety and depression^17^, potentially creating a vicious circle in which each problem exacerbates the others.

The assessment of PsA is complex and multidimensional. It is increasingly common for rheumatologists to include the patient perspective using patient-reported outcomes (PROs) in the evaluation of various domains of the disease and comorbidities, reflecting a paradigm shift in patient assessment. Although some research has explored sleep quality in patients with PsA, it has various limitations. Most studies have had small sample sizes, failed to assess sleep quality considering the inflammatory process together with the psychological well-being of patients, and have not described any use of hypnotics (sleep medication) to manage sleep**.** Further, research to date has not provided data on the relationship of sleep quality with axial forms of the disease or measures of disease activity. In this context, the objective of this study was to assess sleep quality in patients with PsA and its relationship with clinical characteristics, disease activity, functioning, impact of the disease, fatigue and psychological status.

## Methods

### Study population

We carried out a single-centre cross-sectional study at a tertiary hospital in Spain including consecutive patients over 18 years of age seen in rheumatology consultations between January 2021 to December 2021 who met the ClASsification for Psoriatic ARthritis criteria^18,19^.

### Clinical variables

In our clinic, patients with PsA are routinely assessed by taking a detailed medical history, conducting a complete physical examination, gathering PROs and performing laboratory tests every 3 to 6 months. In this clinic, we record demographic data (age and sex) and collect and update information regarding smoking status (smoker/former smoker/never smoker) and the number of cigarettes smoked measured in pack years^19^, use of conventional synthetic disease modifying anti-rheumatic drugs (DMARDs) (methotrexate, sulfasalazine or leflunomide), as well as targeted synthetic or biologic DMARDs, PsA and psoriasis duration, body mass index (BMI).

The level of physical exercise using the International Physical Activity Questionnaire (IPAQ)^20,21^. The aforementioned questionnaire assesses physical activity based on three characteristics: intensity (low, moderate, vigorous), frequency (days per week) and duration (minutes per day). The activity is recorded in metabolic equivalent of tasks (METS). To estimate the number of METs, we multiplied the MET score for the type of activity (3.3 for low, 4 for moderate and 8 for vigorous) by the number of days a week the activity is done and by the minutes spent doing the activity per day.

Among the clinical forms of the disease, axial psoriatic arthritis was defined as inflammatory lower back pain with radiographic damage (at least grade 2 radiographic sacroiliitis as per New York criteria and/or presence of syndesmophytes)^22,23^.

We explored tender joint count (TJC), swollen joint count (SJC), entheseal involvement using the Maastricht Ankylosing Spondylitis Enthesitis Score (MASES)^24^ modified for PsA to include the plantar fascia, with scores ranging from 0 to 15 (mMASES)^25^ and current or history of dactylitis in patients with peripheral forms and a combination of both assessments was used for mixed forms.

The extent of the psoriasis was assessed using the Psoriasis Area Severity Index (PASI)^26^ and item 3 (concerning skin problems) of the Psoriatic Arthritis Impact of Disease (PsAID) questionnaire^27^.

Among the comorbidities considered, we assessed the presence of fibromyalgia^28^. The study was approved by the Ethics Committee of the Hospital Universitario de Salamanca (EO 20/19). All research was performed in accordance with local guidelines/regulations (Castilla y León, Spain) and with the Declaration of Helsinki. All participants and/or their legal guardians gave written informed consent before inclusion in the study and consented to the publication of the results.

### Indices, questionnaires and PROs

#### Disease activity, functioning, perceived pain and disease activity, and disease impact

##### Disease activity

In patients with peripheral involvement, disease activity was assessed using the Disease Activity Index for PSoriatic Arthritis (DAPSA)^29^, while in those with axial involvement, it was assessed using the Ankylosing Spondylitis Disease Activity Score with C-reactive protein (ASDAS-CRP)^30^, the Bath Ankylosing Spondylitis Disease Activity Index (BASDAI) and BASDAI item 2 which relates to axial pain^31^.

##### Functioning

Functioning was measured using the Health Assessment Questionnaire (HAQ)^32^ and the Bath Ankylosing Spondylitis Functional Index (BASFI)^33^.

##### Perceived pain and disease activity

The level of pain and disease activity as reported by the patient using visual analogue scales (VAS).

##### Disease impact

The impact of the psoriatic disease was assessed using the Psoriatic Arthritis Impact of Disease (PsAID) questionnaire^27^.

#### Anxiety, depression, fatigue and sleep

##### Anxiety and depression

Anxiety and depression were assessed with the anxiety and depression subscales of the Hospital Anxiety and Depression Scale (HADS). Using this 14-item self-report instrument developed by Zigmond and Snaith^34^, patients rate their symptoms on a Likert type scale. Seven of the 14 items concern depressive symptoms (HADS-D) and the other seven symptoms of anxiety (HADS-A).

##### Fatigue

Fatigue was assessed with a Functional Assessment of Chronic Illness Therapy (FACIT) scale, specifically, the FACIT-fatigue scale, which has been validated for PsA^35^ and consists of 13 items assessing self-reported fatigue and its impact on activities of daily living and functioning. Items are rated on a 5-point Likert type scale from 0 to 4 yielding a total score between 0 and 52, higher scores indicating less fatigue. Permission was obtained from the FACIT.org for the use of the questionnaire in this study.

##### Sleep

Sleep quality was assessed with a specific tool for measuring sleep quality, the *Pittsburgh Sleep Quality Index* (PSQI)^36^. Using this 19-item self-report instrument patients assess their quality of sleep over the previous 30 days. The PSQI explores seven domains: subjective sleep quality, sleep latency, sleep duration, habitual sleep efficiency, sleep disturbances, use of sleep medication, and daytime dysfunction. Each domain is scored on a range between 0 and 3 and these scores are summed to obtain a global score ranging between 0 and 21 points, 0 reflecting no difficulties at all and 21 serious difficulties in all domains assessed.

### Statistical analysis

Quantitative variables are reported as means and standard deviations and categorical variables as numbers and percentages. Comparisons between groups were carried out using the Student’s t test for normally distributed quantitative variables and the Mann–Whitney U test for ordinal variables or non-normally distributed quantitative variables. Comparisons between more than two groups were performed using one-factor analysis of variance for normally distributed quantitative variables and the Kruskal–Wallis H test for ordinal variables or non-normally distributed quantitative variables. Correlations between quantitative variables were assessed with Spearman’s correlation coefficient. P values < 0.05 were considered statistically significant.

Bivariate correlations were calculated between PSQI values and the following: demographic characteristics (age, BMI, smoking pack years, level of exercise), mMASES, skin manifestations, disease activity (DAPSA and individual DAPSA component scores, activity and pain VAS score, TJC, SJC and C-reactive protein (CRP) level, ASDAS-CRP, BASDAI total and BASDAI item 2 score, functioning (HAQ and BASFI scores), impact of the disease (PsAID) and comorbidities, namely, fatigue (FACIT-F), anxiety (HADS-A) and depression (HADS-D).

The multiple regression model was constructed with the PSQI as the dependent variable and as independent variables those that according to the literature had been related to sleep quality (pain VAS, TJC, RCP, HADS-A, HADS-D and FACIT-F) adjusted for sex and presence of fibromyalgia^2,12,37–40^.

Missing data was less than 3%.

This analysis was performed with IBM SPSS Statistics for Windows, Version 23.0.

### Ethics approval and consent to participate

The study was approved by the Ethics Committee of the Hospital Universitario de Salamanca (EO 20/19).

## Results

### Patient baseline characteristics

Table 1 summarises the characteristics of the 247 patients included in the study. Over half of them were men (55%), the overall mean age was 52.4 ± 11.7 years, and the mean duration of PsA and psoriasis were 9.7 ± 7.4 years and 19 ± 14.4 years respectively. Regarding smoking habits, 73 (30%) were non-smokers, 102 (41%) former smokers and 72 (29%) smokers with a 20.4 ± 24.5 pack year history of smoking. Patients were in the overweight range of BMI 27.2 ± 5.0 kg/m^2^ and had a physical activity level of 616.4 ± 841.0 METs.

**Table 1:** Demographic, clinical and disease-related characteristics of patients with psoriatic arthritis.

Total	n = 247
	Mean ± standard deviation or number (%)
Men	137 (55)
Age (years)	52.4 ± 11.7
Duration of PsA	9.7 ± 7.4
Duration of psoriasis	19.0 ± 14.4
Smoking status	
Smoker	72 (29)
Former smoker	102 (41)
Non-smoker	73 (30)
Smoking pack years	20.4 ± 24.4
BMI (kg/m^2^)	27.2 ± 5.0
Physical exercise (METs)	616.4 ± 841.0
Clinical form	
Peripheral	171 (69)
Mixed	63 (26)
Axial	13 (5)
Conventional synthetic DMARDs	122 (49)
Methotrexate	82 (33)
Sulfasalazine	31 (12)
Leflunomide	9 (4)
Targeted synthetic DMARDs	5 (2)
Biological DMARDs	55 (22)
TNF inhibitor	29 (12)
Other	10 (4)
DAPSA^a^	11.1 ± 6.6
Pain VAS^a^	4.0 ± 2.71
Activity VAS^a^	4.1 ± 0.5
TJC^a^	1.5 ± 2.0
SJC^a^	1.5 ± 1.4
C-reactive protein (g/l)	0.3 ± 0.5
HAQ^a^	0.5 ± 0.5
mMASES	1.4 ± 2.4
Dactylitis	40 (16)
ASDAS-CRP^b^	2.0 ± 0.9
BASDAI^b^	3.7 ± 2.2
BASDAI (item 2)^b^	2.8 ± 2.4
BASFI^b^	3.2 ± 2.7
PASI	1.7 ± 2.7
PsAID	3.2 ± 2.2
HADS anxiety	6.1 ± 4.0
HADS depression	4.3 ± 3.7
FACIT-fatigue	36.6 ± 11.1
Fibromyalgia	11 (4.5)

BMI: body mass index**,** TJC: Tender Joint Count, SJC: Swollen Joint Count, ASDAS-CRP: Ankylosing Spondylitis Disease Activity Score with C-reactive protein; BASDAI: Bath Ankylosing Spondylitis Disease Activity Index; BASDAI (item 2): Bath Ankylosing Spondylitis Disease Activity Index item related to axial pain. BASFI: Bath Ankylosing Spondylitis Functional Index; DAPSA: Disease Activity Index for PSoriatic Arthritis; DMARDs: disease-modifying antirheumatic drug; FACIT: Functional Assessment of Chronic Illness Therapy; HADS: Hospital Anxiety and Depression Scale; HAQ: Health Assessment Questionnaire; mMASES: modified Maastricht Ankylosing Spondylitis Enthesitis; PASI: Psoriasis Area Severity Index; PsA: psoriatic arthritis; PsAID: Psoriatic Arthritis Impact of Disease; PSQI: Pittsburgh Sleep Quality Index; TNF: tumour necrosis factor; VAS: visual analogue scale.

^a^In peripheral and mixed forms (n = 234).

^b^In axial and mixed forms (n = 76).

Regarding the clinical form, a total of 171 (69%) patients were classified as having a peripheral PsA, 13 patients (5%) axial PsA and 63 patients (26%) mixed PsA. The mean TJC and SJC were both 1. Forty patients (16%) had extra-articular musculoskeletal manifestations, in the form of dactylitis, and regarding enthesitis, the mean mMASES score was around 2. A total of 122 (49%) patients were taking conventional synthetic DMARDs, methotrexate in most cases; 55 (22%) were taking a biologic DMARDs, a tumour necrosis factor inhibitor in most cases; and just 5 (2%) were taking a targeted synthetic DMARDs. See Table 1 for more details and other characteristics.

### PSQI questionnaire

Overall, 63% of patients with PsA had poor sleep quality. The highest scores were obtained for items concerning sleep disorders (1.5), duration of sleep (1.5), subjective sleep quality (1.4), sleep latency (1.3) and sleep efficiency (1.1). The use of sleep medication (0.8) contributed less to the total score (Table 2). Notably, just 31% reported using sleep medication three or more times a week, 10% once or twice a week and 12% less than once a week, while the rest (46%) claimed not to use this type of medication at all.

**Table 2:** Components of the Pittsburgh Sleep Quality Index in patients with psoriatic arthritis.

Total	n = 247
Global PSQI score (0–21)	8.5 ± 4.8
PSQI ≥ 6, n (%)	156 (63.1)
Subjective sleep quality (0–3)	1.4 ± 0.7
Sleep latency (0–3)	1.3 ± 1.0
Sleep duration (0–3)	1.5 ± 1.0
Habitual sleep efficiency (0–3)	1.1 ± 1.1
Sleep disturbances (0–3)	1.5 ± 0.7
Use of sleep medication (0–3)	0.8 ± 0.7
Daytime dysfunction (0–3)	0.8 ± 0.7

PSQI: Pittsburgh Sleep Quality Index. Results are expressed as mean (m) and standard deviation (SD).

### Relationship of PSQI with demographic and clinical variables

Among demographic and clinical variables, sleep quality was significantly associated with female sex and the presence of enthesitis (mMASES) (Tables 3 and 4).

**Table 3:** Pittsburgh Sleep Quality Index scores as a function of demographic, clinical and treatment variables.

Variable		PSQI score		p
Sex	Female	Male		
	10.0	7.4		** < 0.001**
Clinical form	Peripheral	Mixed	Axial	
	8.6	8.7	7.1	0.36
Smoking status	Smoker	Former smoker	Non-smoker	
	9.4	8.3	8.0	0.27
Biological DMARDs	Yes	No		
	8.4	8.6		0.78
Dactylitis	Yes	No		
	8.6	8.5		0.90
Fibromyalgia	Yes	No		
	13.7	8.3		** < 0.001**

DMARDs: disease-modifying anti-rheumatic drugs; PSQI: Pittsburgh Sleep Quality Index.

Significant values are in bold.

**Table 4:** Coefficients of correlation of PSQI scores with variables and indices, questionnaires and PROs.

Variables	Spearman coefficient*	p
Age (years)	0.00	0.9
BMI (kg/m^2^)	0.01	0.9
Smoking pack years	0.26	**
Physical exercise (METs)	− 0.05	0.7
mMASES	0.27	**
PASI	0.04	0.7
PsAID item 3	0.39	**
DAPSA	0.48	**
Activity VAS	0.30	**
Pain VAS	0.50	**
TJC	0.34	**
SJC	0.17	**
CPR (g/l)	− 0.07	0.6
HAQ	0.44	**
PsAID	0.60	**
ASDAS-CRP	0.48	**
BASDAI	0.52	**
BASDAI item 2	0.61	**
BASFI	0.43	**
FACIT-fatigue	− 0.61	**
HADS anxiety	0.52	**
HADS depression	0.48	**

BMI: body Mass Index, TJC: Tender Joint Count, SJC: Swollen Joint Count, CRP:C-reactive protein, ASDAS-CRP: Ankylosing Spondylitis Disease Activity Score-CRP; BASDAI: Bath Ankylosing Spondylitis Disease Activity Index; BASDAI (item 2): Bath Ankylosing Spondylitis Disease Activity Index item related to axial pain. BASFI: Bath Ankylosing Spondylitis Functional Index; DAPSA: Disease Activity Index for Psoriatic Arthritis; FACIT: Functional Assessment of Chronic Illness Therapy; HADS: Hospital Anxiety and Depression Scale; HAQ: Health Assessment Questionnaire; mMASES: modified Maastricht Ankylosing Spondylitis Enthesitis; PASI: Psoriasis Area Severity Index; PROs: patient reported outcome; PsAID: Psoriatic Arthritis Impact of Disease; PsAID (item 3) Psoriatic Arthritis Impact of Disease item related to skin problems; PSQI: Pittsburgh Sleep Quality Index; VAS: visual analogue scale.

*Spearman’s r, **p < 0.01.

### Relationship of PSQI with disease activity, functioning and disease impact

Sleep quality as measured by PSQI was correlated moderately with peripheral disease activity (DAPSA), its components pain VAS, and weakly with activity VAS, TJC and SJC. No relationship was observed with CRP level (Table 4). Further, sleep quality was moderately correlated with functioning (HAQ) and with the impact of the disease (PsAID) (Table 4). In patients with axial manifestations, sleep quality was correlated with disease activity (ASDAS-CRP and BASDAI and within this index, with item 2 corresponding to inflammatory axial pain) and moderately with axial functioning (BASFI) (Table 4).

### Relationship between PSQI and cutaneous psoriasis

We found no relationship between sleep quality as measured by the PSQI and cutaneous manifestations of psoriasis as measured by the PASI (r: 0.12; p = 0.54). On the other hand, we observed a weak correlation between PSQI score and item 3 of the PsAID, concerning skin problems including itchiness (r: 0.39; p < 0.001) (Table 4).

### Relationship of PSQI with fibromyalgia, fatigue, anxiety and depression

Patients with fibromyalgia had poorer sleep quality (13.7 ± 3.9 vs 8.3 ± 4.7 in patients who did not have this condition; p < 0.001) (Table 3). Further, sleep quality showed a moderate negative correlation with FACIT-fatigue scores (higher scores on this scale indicating less fatigue) and a moderate positive correlation with HADS scores for anxiety and depression (Table 4). In the multiple linear regression model, pain VAS and fatigue were found to be significant (respectively, β: 0.3; p < 0.007; 95% CI 0.09–0.5 and β: − 0.1; p < 0.001; 95% CI − 0.2 to − 0.1; adjusted R2: 0.4. No statistically significant differences were found with RCP (p = 0.7), TJC (p = 0.07), HADS-A (p = 0.06) and HADS-D (p = 0.1).

## Discussion

Our study confirms the poor sleep quality of patients with PsA and shows its relationship with pain, fatigue, anxiety and depression. To date, few studies have assessed sleep quality in PsA patients using validated questionnaires such as the PSQI^2,12^. In our study, we found a decline in sleep quality in almost two-thirds (63.15%) of patients, similar to the 67.6% described by Krajewska^2^ and somewhat lower than the rates of 84–85% in the cohorts analysed by Gezer^12^ and Wong^41^. In our cohort, the mean global PSQI score was 8.58, slightly lower than values reported by Krajewska^2^, Gezer^12^ and Wong^41^ (scores of 9.32, 9.70 and 9.24 respectively), though higher than scores in the controls of these cohorts (of between 4 and 5).

Lower sleep quality in our patients with PsA was associated with sleep disorders, duration, subjective quality, latency and efficiency, similar to in the aforementioned studies. Also consistent with previous studies, the use of sleep medication was relatively uncommon^2,12,41^. Regarding disease activity, functioning and disease impact, we observed that poorer sleep quality was associated with greater peripheral activity (DAPSA), poorer functioning (HAQ) and greater impact of PsA (PsAID).

One of the main factors associated with poorer sleep quality was pain, a relationship which was also found in the cohorts of Krajewska and Gezer^2,12^. Nonetheless, it is not easy to establish a causal relationship between pain and sleep quality. Some studies^42^ have described apparent bidirectionality in the relationship, with pain leading to a decline in sleep quality but also sleep dysfunction resulting in a worsening of pain. The mechanisms of interaction between sleep quality and pain could be explained from three different perspectives^43^. First, from a neurobiological perspective, sleep interruption, fragmentation or restriction causes hyperalgesia and may interfere with analgesic treatments involving opioidergic and serotonergic mechanisms of action^44^. Secondly, from a psychological perspective, sleep disturbance reduces the pain threshold and amplifies pain signals, resulting in hyperalgesia and more negative emotions focused on pain, forming a negative feedback loop. Thirdly, we should not forget the inflammatory mechanism of axial pain in PsA patients, which again links the idea of a decline in sleep quality with inflammatory activity. In relation to this, sleep quality in axial forms was also associated with disease activity (ASDAS-CRP, BASDAI), axial inflammatory pain and functioning (BASFI). To our knowledge, no previous studies have linked sleep quality with axial manifestations of PsA. In particular, Gezer et al.^12^ did not find any associations between sleep quality and axial signs and symptoms of PsA (sacroiliitis, spondylitis), though in patients with ankylosing spondylitis, sleep quality was associated with inflammatory axial pain, as well as BASDAI and BASFI scores^43^.

Although in our univariate analysis sleep quality as measured by PSQI score was associated with TJC, this potential relationship was not significant in the linear regression model. In patients with psoriasis, the studies of Callis Duffin^17^ and Strober^37^ have previously linked the coexistence of arthritis and psoriasis with poorer sleep quality. On the other hand, neither of these studies assessed whether the worsening in sleep quality was proportional to the intensity of the inflammation, as measured by TJC or CRP levels. Findings in other series of patients with PsA have differed markedly. PSQI scores were not found to be related to TJC by either Gezer et al.^12^ or Krajewska et al.^2^, while both studies found an association between these scores and blood CRP levels. Such differences might be explained by the small sample sizes in these studies. On the other hand, Wong et al.^41^ did find a correlation with TJC but did not assess the correlation with CRP. Further, these authors did not specify whether the variables fatigue or anxiety were included in the multivariate analysis.

In our study, no association was found between psoriasis severity (as measured by PASI score and patient´s subjective perception) and sleep quality. Although such an association has been described in cohorts of patients with psoriasis, it has not been confirmed in other studies in patients with PsA^10,17^. The less severe cutaneous involvement in patients with PsA than those with patients with an exclusively cutaneous disease may explain these results.

In this study, we have confirmed sleep quality to be closely related to fatigue, anxiety and depression. Other studies carried out in patients with PsA have obtained similar results. In these patients, fatigue is more closely associated with emotional than inflammatory characteristics^38^. One of the consequences of poor sleep quality is fatigue. Additionally, it has been demonstrated that the fatigue associated with anxiety and depression may affect sleep quality. Therefore, sleep, fatigue, anxiety and depression should be considered in terms of a circular relationship^39,40^.

At this stage, we should recognise the strengths and limitations of our study. Concerning strengths, first, it was based on a relatively large sample, most published studies being limited by the small number of patients included. Second, the instruments we have used to measure sleep quality (PSQI), fatigue (FACIT-F), anxiety and depression (HADS) have been validated for use in Spain. Lastly, this is the first study to find a link between axial manifestations of the disease and sleep quality.

Regarding limitations, the cross-sectional nature of the study means that we are able to establish associations, but not causal relationships between the factors identified and sleep quality. Second, we did not include patients randomly, but we believe that the inclusion of consecutive patients for a year allowed us to obtain reasonable representativeness, as patients are seen routinely at 3- to 6-month intervals, and hence, all patients should have been assessed at least once during the recruitment period.

## Conclusions

In conclusion, poor sleep quality is common in patients with PsA, although nearly half of patients do not take any sleep medication. In our series, while sleep quality was not associated with cutaneous involvement, it was related to axial manifestations of the disease. Emotional factors (fatigue, anxiety) seem to be more relevant than inflammatory factors in terms of sleep quality. This circular relationship between sleep quality, emotional disorders and disease activity underlines the need to take a multidisciplinary approach to PsA. The detection and management of comorbidities that may influence the activity of patients with PsA is a key aspect in the development of precision medicine to improve the social functioning of patients.

## Data Availability

All data generated or analysed during this study are included in this published article.

## Abbreviations

ASDAS-CPR: Ankylosing Spondylitis Disease Activity Score-C-reactive
BASDAI: Bath Ankylosing Spondylitis Disease Activity Index
BASFI: Bath Ankylosing Spondylitis Functional Index
BMI: Body mass index
CASPAR criteria: ClASsification for Psoriatic ARthritis criteria
cDAPSA: Clinical Disease Activity Index for PSoriatic Arthritis
CRP: C reactive protein
DAPSA: Disease Activity Index for PSoriatic Arthritis
DMARDs: Disease modifying anti-rheumatic drugs
FACIT: Functional Assessment of Chronic Illness Therapy
FACIT-F: Functional Assessment of Chronic Illness Therapy
HADS: Hospital Anxiety and Depression Scale
HADS-A: Hospital Anxiety and Depression Scale-anxiety
HADS-D: Hospital Anxiety and Depression Scale-depressión
HAQ: Health Assessment Questionnaire
IPAQ: International Physical Activity Questionnaire
MASES: Maastricht Ankylosing Spondylitis Enthesitis Score
mMASEs: Modified Maastricht Ankylosing Spondylitis Enthesitis Score
PASI: Psoriasis Area Severity Index
METS: Metabolic equivalent of tasks
PROs: Patient-reported outcomes
PsA: Psoriatic arthritis
PsAID: Psoriatic Arthritis Impact of Disease (PsAID)
PSQI: Pittsburgh Sleep Quality Index
SD: Standard deviation
SJC: Swollen joint count
TJC: Tender joint count
TNF: Tumour necrosis factor
VAS: Visual analogue scales
